# Perceived COVID-19 Severity, Risk of Infection, and Prevention Self-Efficacy in Saudi Arabia During Lockdown: A Population-Based National Study

**DOI:** 10.1007/s44197-022-00083-z

**Published:** 2023-01-21

**Authors:** Rajaa Al-Raddadi, Nezar Bahabri, Zeyad AlRaddadi

**Affiliations:** 1 Faculty of Medicine, Department of Community Medicine, King Abdulaziz University, Jeddah, Saudi Arabia; 2 Samir Abbas Hospital, Jeddah, Saudi Arabia; 3 King Faisal Specialist Hospital and Research Center, Stem Cell and Tissue Engineering Program, Riyadh, Saudi Arabia

**Keywords:** COVID-19, Risk perception, Health belief model, Preventive behavior, Saudi

## Abstract

Evidence shows that the risk perception of humans can significantly affect their response to a threat. This population-based, cross-sectional study explored the determinants of perceived disease seriousness, perceived disease infectiveness, and perceived prevention self-efficacy of coronavirus disease (COVID-19), during the lockdown period, using the health belief model for preventive behavior (HBMPB) among 4423 adults in Saudi Arabia from 13 provinces. Multivariate binary regression was used to analyze the independent factors of three risk perception variables and to measure their effect on adherence to preventive measures. Overall, COVID-19 seriousness was perceived to be higher than that of diabetes and lower than that of a heart attack, while its infectiousness was perceived to be high by 75.3% of the participants. Furthermore, 66.6% had a low perception of their prevention self-efficacy. The HBMPB showed independent effects of all three risk perception parameters on adherence to the preventive measures, including perceived seriousness (Odd’s ratio [OR] = 1.26; 95% confidence interval [95% CI] = 1.01–1.56), infectiousness (OR = 1.90; 95% CI = 1.52–2.38), and prevention self-efficacy (OR = 1.51; 95% CI = 1.20–1.91). Authorities should maintain an optimal level of communication on the COVID-19 risk, communicate more about the virus’ cycle and the disease to demystify the rationale of the preventive measures, and enhance confidence in their efficiency.

**Supplementary Information:**

The online version contains supplementary material available at 10.1007/s44197-022-00083-z.

## Introduction

By the end of 2019, the world was hit by a novel coronavirus pandemic, which is responsible for a highly infectious severe acute respiratory syndrome called coronavirus disease (COVID-19). The number of cases has rapidly increased, and the World Health Organization (WHO) declared it a pandemic on March 11, 2020. Since then, this outbreak has monopolized the world’s attention and efforts to control the spread of the virus and mitigate its health, economic, and overall impact [1–3]. As of the time of this study, knowledge about the disease was limited; vaccines against the causative agent were unavailable; and the effectiveness of the treatments was limited and under evaluation [4, 5].

Therefore, to control the spread of the virus and slow down the transmission and infection rate, prevention and barrier measures have been highly recommended by governments, healthcare professionals, and public institutions. Aggressive infection control measures have been implemented worldwide to break the human–human infection cycle, including restrictive measures with burdensome economic and psychological costs, both on individuals and nations [2, 6–10]. To date, approximately 18 months after the onset of the COVID-19 pandemic and after the start of the global vaccination campaign, protective and restrictive measures are still applied at various levels across countries, especially with the intermittent surges of the pandemic and emergence of mutated strains of the virus [11–14]. This highlights the necessity of maintaining a high level of public awareness and adherence to ongoing and adaptive prevention strategies.

Public behavior is a critical determinant of the effectiveness of infection prevention strategies. Evidence shows that the risk perception of humans can significantly affect their response to a threat. Previous studies conducted during the influenza, SARS, and other outbreaks showed that the populations and individuals with higher levels of risk perception were more likely to be engaged in infection prevention behaviors to reduce the risk of infection and disease transmission [15–18]. During the current COVID-19 pandemic, early population-based surveys in Middle Eastern countries, such as Qatar, Egypt, and Jordan, showed variable levels of risk perceptions and self-efficacy to cope with the crisis, which predicted the levels of adherence to preventive measures [19, 20]. On the other hand, risk perception may be independently associated with several sociodemographic and intrapersonal factors, which include cognitive and emotional factors such as culture, values, knowledge, beliefs, and opinions [21–23]. Understanding how COVID-19 risk is perceived among the population and the associated behaviors is essential for preparing an effective plan for risk communication.

This study aimed to predict public behavior towards COVID-19 preventive measures in Saudi Arabia using HBMPB [24]. The perceived seriousness and infectiveness of COVID-19 perceived self-efficacy in preventing COVID-19 as well as the associated sociodemographic predictors were analyzed.

Such a model would provide solutions to inform decision makers on communication strategies and preventive policies to best engage the population in the fight against the COVID-19 pandemic.

## Materials and Methods

### Design 

This was a population-based, cross-sectional, analytical study involving adults. Data were collected during the lockdown period of June–July, 2020.

### Population and Sampling 

All adults aged 18 years and above residing in Saudi Arabia during the study period were eligible. Saudi Arabia is divided into 13 administrative provinces. A stratified sampling method was used to include a representative number of participants from each province. The sample size was calculated within each stratum, using open Epi to detect an unknown proportion (*p* = 0.50) of participants with a high perceived risk of COVID-19 infection, using an 80% statistical power, 5% margin error, and 95% confidence interval (95% CI). The estimated sample size for each province was 384, resulting in a total target sample size of 4992.

### Data Collection 

A structured questionnaire based on the HBMPB theory, as adapted by Champion and Skinner (2008), was used. The theory provides an explanative model for prevention behaviors against a given condition, based on intrapersonal factors in interaction with sociodemographic and other factors. Intrapersonal factors consist of multidimensional cognitive perceptions towards the seriousness of a given condition, individual susceptibility to the condition, benefits from prevention, barriers to prevention, self-efficacy in prevention, and cue to action [24]. The questionnaire was divided into sections include consent form, which contained a short summary of the study aim, relevance, and importance, with a reminder of the participants’ ethical rights of voluntary participation, unconditioned withdrawal, confidentiality, sociodemographic data, exposure to and impact of COVID-19, including prior COVID-19 diagnosis, history of COVID-19 diagnosis and mortality in relatives, and economic impact of COVID-19.

For perceived seriousness of COVID-19, two approaches were used: (a) a non-comparative approach using a numeric scale from 10 to 100 to rate the perception of COVID-19 seriousness (1 question); and (b) a comparative approach by referring to three other diseases including diabetes, heart attack, and seasonal flu—a 4-level Likert-type scale (1: never dangerous, 2: not dangerous, 3: somewhat dangerous, and 4: dangerous) was used.

The questions that used to assess perceived risk of infection and related psychological state: (1) a 7-level (1–7) Likert-type question on perceived risk (1: null, 7: inevitable) of being infected with COVID-19 in the absence of preventive measures; (2) a question on the level of concern about contracting COVID-19 rated using a 5-level scale (from 1 = not concerned at all to 5 = very concerned); and (3) two questions regarding the frequency of anxiety and obsessive fear towards COVID-19 using a 4-level scale (from 1 = never to 4 = almost every day).

Perceived self-efficacy in preventing COVID-19 was assessed using 5-level confidence scale (from 1 = not confident to 5 = very confident) was used to assess this.

Adherence to preventive measures, which was the outcome of interest (preventive behavior), was assessed using an 11-item dichotomous (not adherent vs. adherent) scale for 11 preventive measures, such as handwashing, masking, and social distancing. The adherence scale was used to calculate scores for the optimal and sub-optimal levels of adherence.

Cognitive barriers to adherence to preventive measures: an 8-item dichotomous (agree vs. disagree) scale was used to assess whether statements such as “I am not exposed” or “Preventive measures are time consuming” could prevent adherence to preventive measures. The cognitive barrier scale was used to calculate the scores for high and low cognitive barriers.

Need for information regarding COVID-19: a 4-item dichotomous (no [I do not need] vs. yes [I need]) scale was used to assess whether the participant needed to be informed about transmission or incubation, symptoms, or prevention. The information scale was used to calculate the scores for optimal and sub-optimal knowledge levels.

The questionnaire was developed in English, translated into Arabic, and then back-translated into English independently by two experienced translators. The back-translated version was compared with the original English version, and any discrepancies were resolved by discussion until a consensus was arrived at.

Face and content validity of the questionnaire was assessed by two independent public health specialists, each has provided recommendations regarding the relevance and formulation of each item. The updated version was resent for review by both specialists, after which the final version was edited. In addition, the reliability was analyzed for the three scales by calculating Cronbach’s alpha.

The questionnaire was edited for online administration using SurveyMonkey. The e-link of the questionnaire was attached to an introductive message on the study aim and importance, which was disseminated electronically via different social media platforms, including WhatsApp, Facebook, Twitter, and Snapchat. The e-link was sent weekly for 4 weeks to increase the participation rate until the desired sample size was attained.

### Missing Data Management

Some participants had missing data on the perceived severity of COVID-19, diabetes, heart attack, and influenza—0.4–0.9% of the overall participants depending on the item. Central values (medians) were used to replace the missing values. Other missing data on adherence to preventive measures (0.1–0.2% depending on the item), cognitive barriers to adherence (0.1–0.8% depending on the item), and need for information (0.7–1.0%) were replaced by the corresponding negative responses, that is, not adherent, disagree, and not needed, respectively. Specifically, the absence of a response led to the assumption that the participant excluded the positive option, since all the variables were dichotomous. No further missing data were found.

### Statistical Methods

The database was automatically imported from SurveyMonkey to the Statistical Package for the Social Sciences (SPSS version 21.0; SPSS, Chicago, Illinois, USA), where it was cleaned and coded for data analysis.

Descriptive statistics were used to summarize the patterns of answers to the different sections of the questionnaire, and the Kolmogorov–Smirnov test was used to test the normality of continuous variables.

The Chi-square test was used to analyze the associations between the different categorical variables, to compare the sociodemographic characteristics between those who participated incompletely and completely, and to analyze factors associated with levels of perceived seriousness, perceived risk of infection, and perceived prevention self-efficacy. The calculated scores are summarized as the mean (95% CI).

The comparative (or relative) perceived seriousness of COVID-19 with the three other conditions (Δ) was analyzed using the Wilcoxon signed-rank test, a paired-sample non-parametric test. Results are presented as the mean difference (mean Δ) between perceived seriousness of COVID-19 and that of the other disease, where positive and higher values indicate a higher seriousness perceived towards COVID-19 compared to the reference disease, and negative and lower values indicate a lower seriousness perceived towards COVID-19 compared to the reference disease. Further, comparative seriousness findings were plotted as a mirrored area chart, where the height of the chart corresponds to the percentage of participants displaying the given values of relative perceived seriousness (Δ) with reference to the given condition.

Multivariate binary regression was used to analyze the independent factors of perceived seriousness, perceived infectiveness, and perceived prevention self-efficacy. Results are presented as odds ratios (ORs) with 95% CIs. Subsequently, a multivariate binary regression model was tested to measure the effect of the three risk perception parameters on the binomial variable of adherence to preventive measures. A *p* value < 0.05 was considered statistically significant.

Ethical approval was obtained from the central institutional review board committee of the Ministry of Health (serial number 20-#814E). All participants were asked to consent at the beginning of the questionnaire.

## Results

**Fig. 1:**
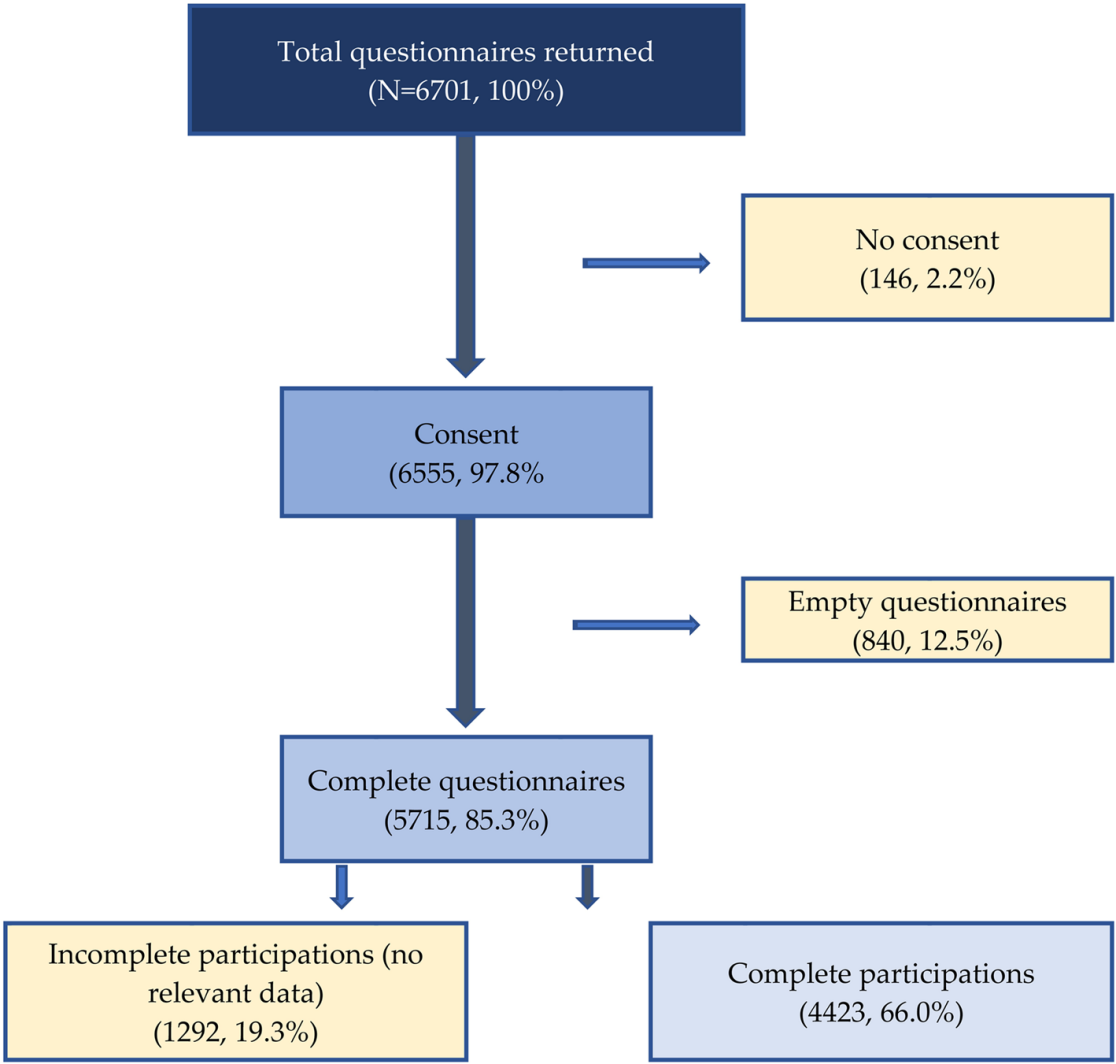
Participant selection flowchart

Of the total 6701 returned questionnaires, 5715 (85.3%) were answered; 1292 were incomplete and contained no relevant outcome data and 4423 were complete (response rate = 66.0%) (Fig. 1).

The sociodemographic characteristics of individuals with complete and incomplete survey are shown in Table 1. The participants who incomplete the survey had a higher female ratio (*p* = 0.002), a higher percentage of younger persons (*p* < 0.001), and lower socioeconomic (*p* ≤ 0.001) and educational levels (*p* < 0.001), in addition to varying marital and professional statuses. Furthermore, individuals with a prior COVID-19 diagnosis were more likely to complete the survey (OR = 1.81, 95% CI = 1.43–2.30; *p* < 0.001) (results not presented in tables or figures).

**Table 1:** Characteristics of the complete versus incomplete surveys

Parameter	Category	Surveys				*p* value
		Incomplete (*N* = 1292)		Complete (*N* = 4423)		
		*N*	%	*N*	%	
Sex	Male	349	27.0	1399	31.6	0.002
	Female	943	73.0	3024	68.4	
Age (years)	< 20	156	12.1	269	6.1	< 0.001
	20–29	294	22.8	1200	27.1	
	30–39	245	19.0	980	22.2	
	40–49	274	21.2	1062	24.0	
	50–59	215	16.6	658	14.9	
	60 +	108	8.4	254	5.7	
Nationality	Saudi	1140	88.2	3921	88.7	0.680
	Non-Saudi	152	11.8	502	11.3	
Marital status	Single	428	33.1	1495	33.8	0.004
	Married	760	58.8	2676	60.5	
	Divorced	70	5.4	193	4.4	
	Widowed	34	2.6	59	1.3	
Province (13)	Najran (min)	8	0.6	26	0.6	0.596
	Makkah (max)	394	30.5	1371	31.0	
Family income (SAR)	< 5000	390	30.2	1072	24.2	< 0.001
	5000–10,000	307	23.8	895	20.2	
	10,000–15,000	251	19.4	941	21.3	
	15,000–20,000	161	12.5	690	15.6	
	> 20,000	183	14.2	825	18.7	
Occupation	Healthcare worker	54	4.2	282	6.4	< 0.001
	private sector	129	10.0	560	12.7	
	Governmental sector	349	27.0	1360	30.7	
	Retired	157	12.2	452	10.2	
	Unemployed	284	22.0	872	19.7	
	Student	280	21.7	802	18.1	
	Other	39	3.0	95	2.1	
Educational level	Primary	25	1.9	28	0.6	< 0.001
	Intermediate	71	5.5	15	2.6	
	Secondary	347	26.9	696	15.7	
	Diploma	136	10.5	396	9.0	
	Bachelor	613	47.4	2684	60.7	
	Masters	66	5.1	338	7.6	
	PhD	34	2.6	166	3.8	

### Personal Exposure to COVID-19

After exclusion of the participants who incomplete the survey, 4.9% of the participants had a past COVID-19 infection, and most of them (81.7%) spent their convalescence period at home. Further, 16.2% of the participants declared having at least one relative who was diagnosed with COVID-19, and 2.3% declared having lost a relative from COVID-19 (Table 2).

**Table 2:** Exposure to and impact of COVID-19 (*N* = 4423)

Parameter	Category	Frequency	Percentage (%)
Personal history of COVID-19 infection	Negative	4205	95.1
	Positive	218	4.9
Location during infection	Home	178	81.7
	Hospital	7	3.2
	Quarantine	14	6.4
	Not mentioned	19	8.7
Relative diagnosed with COVID-19	None	3708	83.8
	Yes	715	16.2
Number of concerned relatives	One	240	5.4
	Two	111	2.5
	3–5	198	4.5
	5–10	94	2.1
	> 10	42	0.9
	Unspecified	30	0.7
Lost relative from COVID-19	No	4323	97.7
	Yes	100	2.3
Family income stopped because of COVID-19	No	3709	83.9
	Yes	714	16.1

*COVID-19* coronavirus disease

### Perceived Seriousness of COVID-19

Using a scale of 10–100, COVID-19 seriousness was rated > 50 by 61.0% of the participants; the mean rating was 63.8 (95% CI = 63.1–64.6). With other diseases as a reference, COVID-19 was perceived to be more serious than diabetes (mean Δ = + 0.20, *p* < 0.01) and seasonal influenza (Δ mean = + 0.89, *p* < 0.001), and less serious than heart attack (Δ mean = − 0.31, *p* < 0.001) by the overall participants. Of the total participants, 33.8%, 3.8%, and 72.8% perceived COVID-19 to be more serious than diabetes, heart attack, and seasonal flu, respectively. Meanwhile, 49.5%, 65.0%, and 23.9% perceived that COVID-19 had an equivalent seriousness to that of those conditions (Supplementary Fig. 1). The levels of perceived relative * absolute seriousness were weakly correlated (Pearson’s correlation coefficient, *r* = 0.20–0.31; *p* < 0.001).

### Risk Perception Regarding COVID-19 Infection and Related Psychological Impact

Two-third (75.3%) of the participants agreed that they were at high risk of contracting COVID-19 in the absence of preventive measures; 22.5% of these participants believed that they would surely contract it. On the other hand, only one-third (33.4%) reported being confident or very confident in their ability to prevent themselves from being infected, and 84.9% declared being concerned or very concerned by the issue. The levels of concern and anxiety were relatively high (Table 3).

**Table 3:** Risk perception regarding COVID-19 infection and related psychological impact

Dimension/parameter	Level	Frequency	Percentage (%)	Low vs. high^§^
Perceived risk of infection in absence of preventive measures	Null	115	2.6	24.7
	Extremely low	189	4.3	
	Low	195	4.4	
	Moderate	593	13.4	
	**High**	**1337**	**30.2**	75.3
	**Very high**	**1000**	**22.6**	
	**Certain**	**994**	**22.5**	
Perceived self-efficacy in prevention against COVID-19 infection	Not confident	601	13.6	66.6
	Somewhat confident	1464	33.1	
	Not sure	882	19.9	
	**Confident**	**1009**	**22.8**	33.4
	**Very confident**	**467**	**10.6**	
Concern about contracting COVID-19	Not concerned at all	52	1.2	15.2
	Not concerned	66	1.5	
	Somewhat concerned	551	12.5	
	**Concerned**	**1450**	**32.8**	84.9
	**Very concerned**	**2304**	**52.1**	
Anxiety regarding COVID-19	Never	748	16.9	58.0
	Seldom	1817	41.1	
	**Often**	**832**	**18.8**	42.0
	**Almost every day**	**1026**	**23.2**	
Uncontrollable fear of contracting COVID-19	Never	1421	32.1	69.1
	Seldom	1636	37.0	
	**Often**	**642**	**14.5**	30.9
	**Almost every day**	**724**	**16.4**	

^§^Dichotomized variables, including low (italics) versus high (bold) levels. *COVID-19* coronavirus disease

### Adherence to Preventive Measures

Adherence rates were very high (93.8–98.9%) for all the assessed measures, except for the intake of immunity-reinforcing food (78.9%) and physical exercise (58.0%) (Supplementary Fig. 2). Hence, the overall adherence level showed a right-skewed histogram with a mean adherence score of 10.05 (95% CI = 10.01–10.08) out of 11, with a range of 0–11. Considering immunity-reinforcing food and physical exercise as optional measures, 91.0% of the participants had an optimal level of adherence (score = 9–11), while 9.0% had a sub-optimal level of adherence (score < 9).

The most evoked barrier to adherence to preventive measures was “nobody follows the measures” (30.4%), followed by “preventive measures are time consuming” (23.0%) and “I do not get ill” (22.9%) (Supplementary Fig. 3). The level of cognitive barriers, indicated by the number of cognitive barriers to adherence to prevention measures, showed a left-skewed histogram with a mean score of 1.18/8 (95% CI = 1.14–1.22).

### Need for Knowledge About COVID-19

Participants displayed a high need for COVID-19-related information in all aspects, including virus incubation (72.2%), prevention (63.0%), transmission (61.4%), and COVID-19 symptoms (59.2%) (results not presented in tables or figures). The mean need-for-information score was 2.56/4 (94% CI = 2.51–2.61). Of the total participants, 31.5% had an adequate (need-for-information score = 0 or 1) level of information about COVID-19, while 68.5% had an inadequate level (score = 2–4).

### Factors Associated with Perceived Seriousness, Risk of Infection, and Prevention Self-Efficacy

Perceived seriousness of COVID-19 was significantly lower in males (*p* < 0.001) and previously infected participants (*p* = 0.009), while it was higher in those who had lost a relative from COVID-19 (*p* = 0.002), compared to their respective counterparts. A low perceived risk of infection in the absence of preventive measures was associated with younger age (*p* < 0.001), single status (*p* = 0.006), low income (*p* < 0.001), low education (*p* < 0.001), unemployed or unspecified professional status (*p* < 0.001), prior COVID-19 infection (*p* = 0.022), and sub-optimal knowledge about COVID-19 (*p* = 0.001), compared to their respective counterparts. Low perceived prevention self-efficacy was associated with female sex (*p* < 0.001), middle-aged participants (*p* < 0.001), Saudi nationality (*p* = 0.040), divorced status (*p* < 0.001), unemployed or unspecified professional status (*p* < 0.001), prior COVID-19 infection (*p* = 0.001), and sub-optimal knowledge about COVID-19 (*p* < 0.001) (Table 4).

**Table 4:** Factors associated with perceived seriousness, risk of infection, and prevention self-efficacy

Factor	Category	High perceived seriousness (> 50/100)		High perceived risk of infection		High perceived prevention self-efficacy	
		Rate (%)	*p* value	Rate (%)	*p* value	Rate (%)	*p* value
Sex	Male	54.9	< 0.001*	75.3		41.2	< 0.001*
	Female	63.9		75.3	0.976	29.8	
Age (years)	< 20	65.1	0.062	68.0	< 0.001*	43.5	< 0.001*
	20–29	59.3		73.2		37.8	
	30–39	58.9		73.5		30.9	
	40–49	64.3		78.9		27.8	
	50–59	59.9		78.4		31.9	
	60 +	62.2		77.2		38.6	
Nationality	Saudi	61.2	0.418	75.6	0.151	32.8	0.040*
	Non-Saudi	59.4		72.7		37.5	
Marital status	Single	59.8	0.143	72.2	0.006*	38.9	< 0.001*
	Married	61.8		77.1		30.8	
	Divorced	62.7		74.1		28.0	
	Widowed	49.2		76.3		30.5	
Family income^§^ (SAR)	< 5K	61.9	0.803	69.0	< 0.001*	30.4	0.584
	5K–10K	61.1		75.0		32.8	
	10K–15K	61.8		78.1		31.5	
	15K–20K	59.6		77.5		35.1	
	> 20K	60.6		78.8		33.9	
Occupation	Healthcare worker	57.8	0.105	83.7	< 0.001*	32.6	< 0.001*
	Private sector	56.3		75.9		34.5	
	Governmental sector	63.4		78.2		31.7	
	Retired	60.0		77.4		33.0	
	Unemployed	61.6		68.9		29.9	
	Student	61.1		74.4		40.5	
	Other	64.2		61.1		26.3	
Educational level	Primary	60.7	0.273	64.3	< 0.001*	21.4	0.435
	Intermediate	60.0		61.7		33.0	
	Secondary	62.1		71.8		33.8	
	Diploma	62.1		75.0		34.8	
	Bachelor	61.7		76.5		32.6	
	Masters	56.2		74.9		37.6	
	PhD	54.2		83.1		34.9	
Prior COVID-19 diagnosis	No	61.3	0.153	75.6	0.022*	33.4	0.970
	Yes	56.4		68.8		33.5	
Prior COVID-19 diagnosis in relative	No	61.9	0.009*	75.3	0.885	34.4	0.001*
	Yes	56.6		75.5		28.0	
Loss of a relative from COVID	No	60.7	0.002*	75.2	0.387	33.4	0.769
	Yes	76.0		79.0		32.0	
Family income impacted by COVID-19	No	60.6	0.172	75.8	0.113	32.9	0.147
	Yes	63.3		73.0		35.7	
Level of knowledge	Optimal	59.9	0.299	78.6	0.001*	37.4	< 0.001*
	Suboptimal	61.5		73.8		31.5	

Values are expressed as the rate (%) of high perception level in the given outcome

*Statistically significant difference (*p* < 0.05)

^§^k represented 1000; therefore, 5K represents SAR 5000 and so on

### Regional Differences in the Three Outcomes

**Fig. 2:**
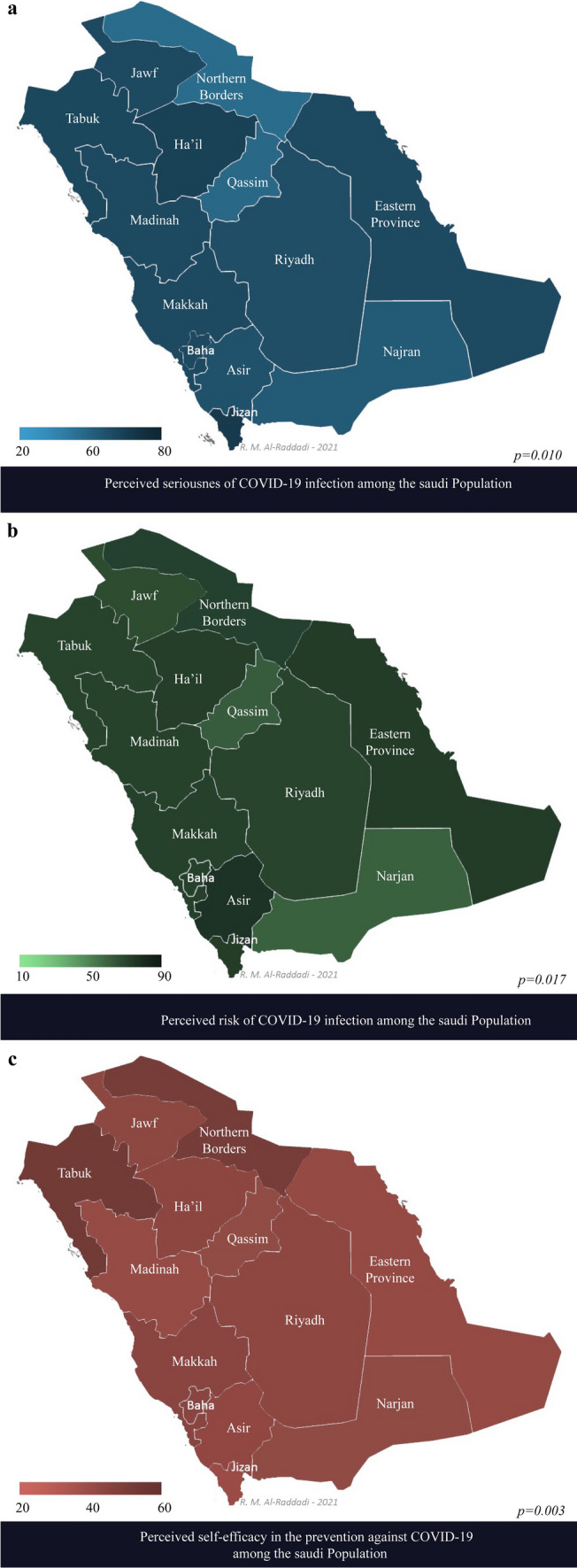
Perceived seriousness of COVID-19 (panel a), perceived risk of infection (panel b) and perceived self-efficacy in prevention against COVID-19 (panel c)

The geographic distribution showed significant differences between the provinces in terms of the three outcomes: high perceived COVID-19 seriousness rates ranged between 45.2% in Northern borders and 69.4% in Jizan (*p* = 0.010) (Fig. 2a), high perceived risk of infection rates ranged between 61.5% in Najran and 83.5% in Asir (*p* = 0.017) (Fig. 2b), and high perceived prevention self-efficacy rates ranged between 27.1% in Jizan and 48.0% in Tabuk (*p* = 0.003) (Fig. 2c).

### Predictors of Perceived Seriousness, Risk of Infection, and Prevention Self-Efficacy

Results of adjusted regression models for perceived seriousness, risk of infection and prevention self-efficacy are depicted in Table 5.

**Table 5:** Predictors of perceived seriousness, risk of infection and prevention self-efficacy

Predictor	Level	OR	95% CI		*p* value
Perceived seriousness					
Gender	Male	0.69	0.61	0.79	< 0.001*
Prior COVID-19 diagnosis	Yes	0.81	0.62	1.07	0.142
Loss of a relative because of COVID	Yes	2.08	1.30	3.31	0.002*
Perceived risk of infection					
Age (years)	< 20	0.60	0.35	1.03	0.065
	20–29	0.82	0.53	1.27	0.373
	30–39	0.83	0.56	1.24	0.364
	40–49	1.07	0.72	1.59	0.744
	50–59	1.06	0.73	1.53	0.756
	60 +	–			
Marital status	Single	0.84	0.44	1.63	0.612
	Married	0.95	0.51	1.78	0.878
	Divorced	0.86	0.43	1.73	0.677
	Widowed	–			
Family income (SAR) ^§^	< 5K	0.77	0.61	0.97	0.026*
	5K–10K	0.91	0.72	1.15	0.430
	10K–15K	1.00	0.79	1.26	0.978
	15K–20K	0.94	0.73	1.21	0.616
	> 20K	–			
Occupation	HCW	–			
	Other private	0.68	0.46	0.99	0.042*
	Other Gov	0.62	0.44	0.88	0.008*
	Retired	0.58	0.37	0.89	0.014*
	Unemployed	0.50	0.35	0.72	0.000*
	Other	0.36	0.21	0.62	0.000*
	Student	0.80	0.54	1.20	0.284
Educational level	Primary	0.55	0.22	1.35	0.191
	Intermediate	0.50	0.28	0.90	0.020*
	Secondary	0.79	0.49	1.26	0.320
	Diploma	0.75	0.47	1.22	0.250
	Bachelor	0.83	0.54	1.27	0.394
	Masters	0.69	0.43	1.12	0.132
	PhD	–			
Prior COVID-19 diagnosis	Yes	0.77	0.57	1.04	0.085
Level of knowledge	Suboptimal	0.80	0.69	0.94	0.005*
Perceived self-efficacy					
Gender	Female	0.58	0.51	0.67	< 0.001*
Age (years)	< 20	–			
	20–29	0.83	0.62	1.12	0.224
	30–39	0.70	0.48	1.01	0.054
	40–49	0.60	0.41	0.88	0.010*
	50–59	0.75	0.50	1.13	0.165
	60 +	1.04	0.64	1.68	0.876
Nationality	Saudi	0.86	0.70	1.06	0.159
Marital status	Single	–			
	Married	0.79	0.64	0.97	0.026*
	Divorced	0.80	0.56	1.15	0.232
	Widowed	0.87	0.47	1.58	0.643
Occupation	HCW	–			
	Other private	1.03	0.75	1.40	0.865
	Other Gov	1.13	0.85	1.50	0.415
	Retired	1.03	0.71	1.49	0.881
	Unemployed	1.03	0.77	1.39	0.836
	Student	1.11	0.80	1.54	0.541
	Other	0.82	0.48	1.40	0.470
Prior COVID-19 diagnosis in relative	Yes	0.76	0.64	0.91	0.003*
Level of knowledge	Suboptimal	0.75	0.65	0.86	< 0.001*

*HCW* healthcare worker, *other Gov*. worker in other governmental sectors, ^§^K represents 1000, therefore, 5K represents SAR 5000 and so on, *OR* odds ratio, *95% CI* 95% confidence interval

*Statistically significant difference (*p* < 0.05)

High perceived seriousness of COVID-19 was independently associated with male sex (OR = 0.69, 95% CI = 0.61–0.79; *p* < 0.001) and the presence of COVID-19-related mortality in relatives (OR = 2.08, 95% CI = 1.30–3.31; *p* = 0.002).

A high perceived risk of infection was independently associated with low family income (< 5 K vs.  > 20 K SAR; OR = 0.77, 95% CI = 0.61–0.97; *p* = 0.026), non-healthcare jobs (ORs ranged between 0.36 and 0.68, depending on the job category; *p* < 0.050), and sub-optimal knowledge about COVID-19 (OR = 0.80, 95% CI = 0.69 to − 0.94; *p* = 0.005). Of note, prior COVID-19 diagnosis was significant at a 10% margin error, associated with an OR of 0.77 (95% CI = 0.58–1.03; *p* = 0.085).

Perceived prevention self-efficacy was independently associated with female sex (OR = 0.58, 95% CI = 0.51–0.67; *p* < 0.001), age of 40–49 years (OR = 0.60, 95% CI = 0.41–0.88; *p* = 0.010), married status (OR = 0.79, 95% CI = 0.64–0.97; *p* = 0.026), prior COVID-19 infection in relatives (OR = 0.76, 95% CI = 0.64–0.91; *p* = 0.003) and sub-optimal knowledge about COVID-19 (OR = 0.75, 95% CI = 0.65–0.86; *p* < 0.001).

**Fig. 3:**
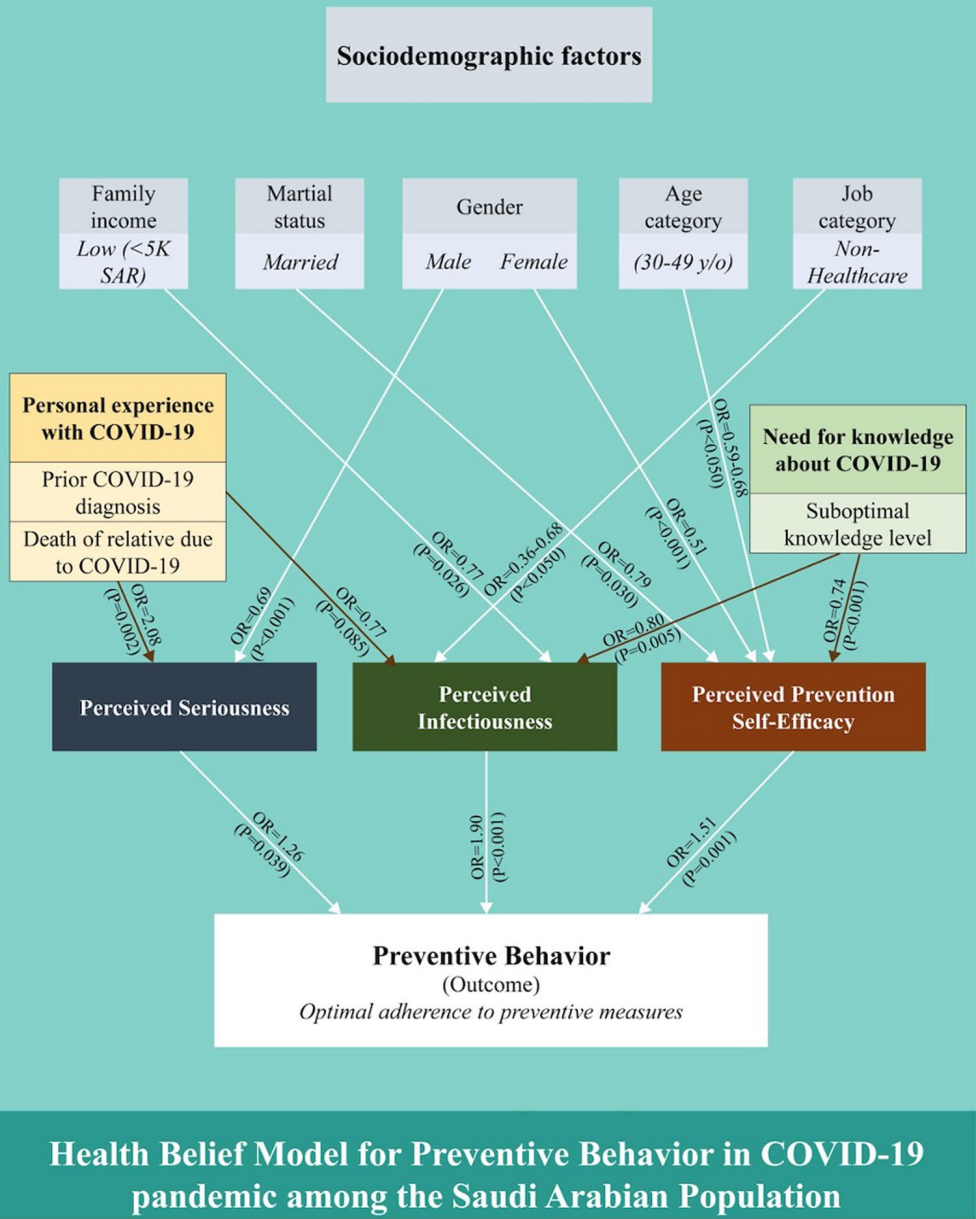
Health belief model for preventive behavior during the COVID-19 pandemic in the Saudi Arabian Population. Values are odds ratios of the given predictor in the adjusted regression equation. The complete data of the regression models are depicted in Table 5

A summary of the study findings according to HBMPB is depicted in Fig. 3. The level of adherence to the preventive measures against COVID-19 was independently associated with perceived seriousness (OR = 1.26, 95% CI = 1.01–1.56; *p* = 0.039), perceived infectiousness or risk of infection (OR = 1.90, 95% CI = 1.52–2.38; *p* < 0.001), and perceived prevention self-efficacy (OR = 1.51, 95% CI = 1.20–1.91; *p* = 0.001).

## Discussion

This study aimed to predict public behavior towards COVID-19 preventive measures using HBMPB. The HBMPB demonstrates the relevance of enhancing public awareness about the seriousness of COVID-19 as a disease, its infectiousness in the absence of preventive measures, and the individuals’ self-efficacy to prevent themselves from contracting the infection.

Syed et al. performed a comparable local study in which the health belief model was used to explore the predictors of adherence to preventive measures against COVID-19 among 668 individuals from all regions of Saudi Arabia. They found that adherence to preventive measures was explained by the perceived susceptibility to the disease, disease severity, perceived benefits of compliance to the measures, and cue to action consisting of the perceived aptitude to practice the preventive measures; this resulted from the Coronavirus Awareness Program of the Ministry of Health. Syed et al. reported the significant contribution of sociodemographic factors in the model, notably age, sex, and marital status; this was similar to our study [25]. Another social media-based study from India involving 2,646 individuals approached the perceived vulnerability to the infection using a tridimensional threat model; this study included vulnerability with respect to the disease progression in time, individual’s intrinsic condition, and residence area. All three dimensions were highly correlated with the perceived disease severity and showed various positive associations with the different preventive measures. Furthermore, the levels of practice of the preventive measures were positively associated with the perceived severity of the disease, as well as with self-confidence in performing the given preventive measures. Remarkably, the authors highlighted that “being informed by any healthcare worker about the COVID-19-appropriate behaviors was more effective in facilitating the adoption of preventive practices among respondents” [26]. Consistent with the previous models, a letter to the editor by Chan et al. explained that, according to self-determination and planned behavior theories, social environments determine the pattern of motivation and behavioral intention of an individual. Authors exposed two patterns of motivation including “autonomous” and “controlled” motivations, advocating that the autonomous mode endorses proactive adherence to healthy behaviors. Such autonomous motivation modes are attainable by the cognitive and psychological integration of the preventive measures to the individual’s scope of “interest, satisfaction, goals, and values”. Based on this reflection, the authors demonstrated the limitation of coercive law enforcement strategies and the detrimental effect of the social environment on individuals’ motivational and behavioral intention on adherence to COVID-19 preventive measures [27].

There is extensive literature on the correlation between risk perception and self-protective behaviors, which is one of the illustrations of cognitive and behavioral theories. In health-related issues, the concept of risk perception represents the factorial result of two aspects: the intrinsic severity or seriousness of the disease and the probability of the person contracting the disease. To elicit the desired outcome behavior, such as adherence to preventive measures, the risk perception theory assumes that individuals use a pragmatic cognitive process to evaluate the risk, seriousness of a disease, and the personal risk to be infected. Subsequently, to implement an effective and cost-effective strategy to achieve the goal, individuals would weigh this risk with their aptitudes to prevent the disease [28–31]. Therefore, risk perception constitutes one of the key communication axes for governments, health authorities, and health providers to elicit positive changes in public behavior, and to enhance their adoption of the implemented protective and preventive measures [29, 31–33]. However, risk perception is subject to several biases and depends on several factors [31, 34]. Furthermore, communication consists of a two-way process, which implies acquiring and evaluating feedback from the targeted groups on their levels of knowledge, and their values and opinions regarding the issue. This approach is critical for both the design and implementation of intelligible and relevant messages, and assessment of the effectiveness of the communication strategy [33, 35]. On the other hand, communications on risk perception should take into consideration that such a strategy is limited by deleterious effects on positivity, death stress, and mood [20].

In this study, we assessed several dimensions that may impact risk perception and found relevant implications. One of the remarkable factors of risk perception identified in this study was the personal experience with COVID-19, which was associated with perceived seriousness (family history of COVID-19-related mortality) and infectiousness (prior COVID-19 diagnosis). The risk of mortality is undeniably a major factor of perceived seriousness of the disease, and was associated with a twofold increase in the probability of perceiving the disease as highly serious in this study. Personal experience with the virus is a transcultural factor that has been reported to be associated with higher risk perception about the disease [36]. The presence of a tragic event in the family, close relatives, or friends produced an amplification effect on risk perception.

The other factor that was independently associated with risk perception, precisely infectiousness, and perceived prevention self-efficacy was the self-reported level of knowledge about the disease. This double association highlights the importance of raising public awareness and knowledge about the COVID-19 cycle of transmission and infectiousness, as well as the rationale for preventive measures. Knowledge has consistently been demonstrated to be associated with risk perception in COVID-19 in several international studies, emphasizing the importance of identifying specific needs for knowledge in a given population [36–40]. In the Saudi population, Al-Hanawi et al. demonstrated a positive relationship between knowledge about COVID-19 and attitude and practice in preventive measures [41]. This highlights the importance of promoting knowledge among the population about COVID-19 to enhance the effective adherence with preventive measures.

Sociodemographic factors may represent the most heterogeneous determinants, not only for risk perception and self-efficacy, but also for the levels of knowledge and other cognitive and psychological factors, in addition to their direct impact on preventive behavior and mechanisms of behavioral change [39, 42–44]. The findings of this study demonstrated a set of independent associations between sociodemographic factors and perceived risk or perceived prevention self-efficacy. For instance, females, middle-aged and low-income individuals had a greater likelihood of perceiving their self-efficacy in prevention to be low. Where female participants had a lower perceived self-efficacy in prevention, they had a higher risk perception compared with males. This consistently reported observation indicates a greater concern among females about contracting the disease or having a severe form. Regarding low-income participants, such observations may be explained by the direct and indirect costs that may be incurred by the implementation of preventive and restrictive measures, such social distancing, hand washing, home quarantine, etc., which may be perceived to be burdensome for low-income persons. Likewise, middle-aged individuals, who represent the most active subpopulation, may find it difficult to adhere to measures such as social distancing and home quarantine. Several studies reported significant associations between COVID-19 risk perception during lockdown and sociodemographic factors. For instance, a population-based Spanish study showed older age, female gender, poor or sub-optimal health status, and having a family member working in healthcare to be positively associated with high perceived risk of COVID-19 [44]. In Italy, the risk of infection was higher in females and correlated significantly with the effect on decision making of endogenous processing of risk perception, which was assessed using the Temperament and Character Inventory [45]. Given that the three perception variables were defined as two-level variables (high vs. low) using respective cutoffs, the model assumed that an adequate level of the individual’s perception should be achieved to elicit the desired preventive behavior, consisting of the optimal adherence to preventive measures. As such, further interventional studies are required to demonstrate the observable effect of improving these three perception parameters on the level of adherence to preventive measures.

To enhance the overall efficacy of preventive strategies for the benefit and safety of the whole population, it is crucial to understand the interplay between the sociodemographic factors and the determinants of risk perception and preventive behavior, and to implement the relevant solutions and support to vulnerable subpopulations.

### Limitations and Strengths

Although this study has a national, population-based design, it has some limitations. First, the online administration of the questionnaire may have induced selection bias by underrepresentation to people who do not use social media while overrepresenting others who share interest and use a specific social media, which was used to disseminate the questionnaire link. In addition, the data collection process failed to balance the representativeness of the sample with respect of gender and region, which resulted in a high female ratio and discrepancy in the proportion of participants across the Kingdom’s provinces. Finally, the Arabic administration of the questionnaire probably excluded non-Arabic speaking individuals living in Saudi Arabia. These limitations should be taken into consideration when generalizing the findings and their implications at the national level.

On the other hand, the present study provided a thorough analysis of sociodemographic factors and exposure with and knowledge about COVID-19, and their interplay with the three dimensions of disease perceptions, along with the impact of all these factors on the preventive behavior. The findings are illustrated in a well-illustrated model that highlights the major targets of communication and policy interventions.

## Conclusions

The Saudi population perceives the seriousness of COVID-19 as being midway between diabetes and heart attack, while one-quarter of them perceived it to be mildly or moderately infectious, and only one-third were confident about their self-efficacy in preventing the disease. The HBMPB showed independent effects of all three risk perception parameters on adherence to the preventive measures, including perceived seriousness, infectiousness, and prevention self-efficacy.

Although remarkably high, the levels of adherence to the different mandatory preventive measures can be further enhanced by increasing the levels of risk perception and self-efficacy. Health authorities and policymakers are recommended to maintain an optimal level of communication regarding the risk of the disease and the efficacy of preventive measures. The specific needs for information should be further explored to improve knowledge about the virus incubation, virus transmission, and disease symptoms; this will demystify the rationale of the preventive measures and enhance confidence in their efficiency. Further interventional studies are warranted to demonstrate the efficacy of risk perception improvement in enhancing protective behavior. Such interventions should eventually target subgroups with a low level of risk perception and low self-efficacy profiles and should explore their specific needs for knowledge and social or psychological support.

## Supplementary Information

Below is the link to the electronic supplementary material.

Supplementary file1 Perceived severity of coronavirus disease (COVID-19) by reference to diabetes, heart attack, and seasonal influenza (TIF 2432 KB)

Supplementary file2 Adherence levels to preventive measures against corona-virus disease (TIF 2245 KB)

Supplementary file3 Cognitive barriers to adherence to preventive measures against coronavirus disease (TIF 2089 KB)

## Data Availability

The datasets are available from the corresponding author on reasonable request.

## Abbreviations

HBMPB: Belief model for preventive behaviors
WHO: World Health Organization
