# Supplementary figures and images for: Perceived COVID-19 Severity, Risk of Infection, and Prevention Self-Efficacy in Saudi Arabia During Lockdown: A Population-Based National Study
Source: J Epidemiol Glob Health. 2023 Jan 21;13(1):32–46. doi: 10.1007/s44197-022-00083-z (PMC9867541; doi:10.1007/s44197-022-00083-z)

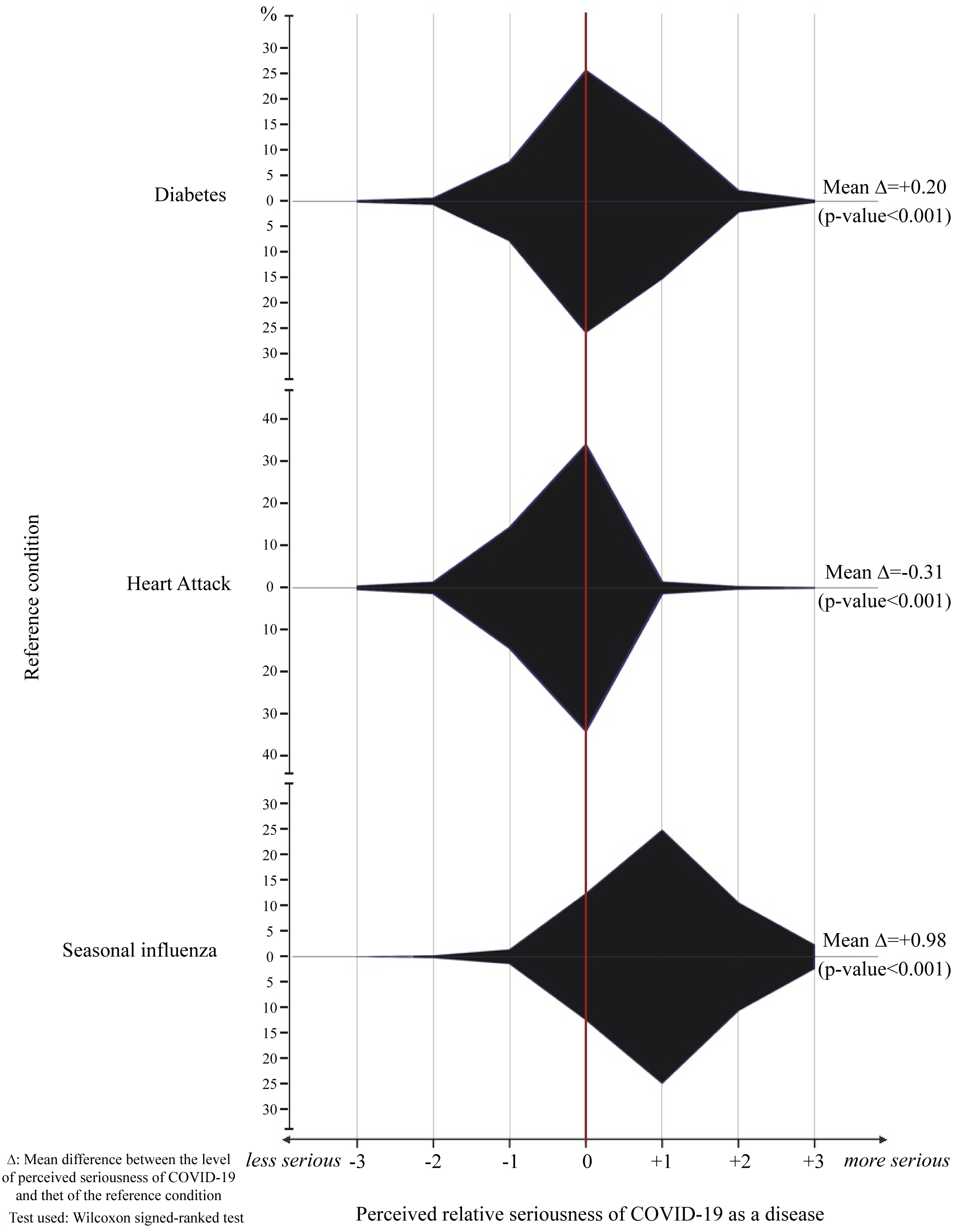

Supplement: Supplementary file 1 — Supplementary file1 Perceived severity of coronavirus disease (COVID-19) by reference to diabetes, heart attack, and seasonal influenza (TIF 2432 KB) [file 44197_2022_83_MOESM1_ESM.tif]

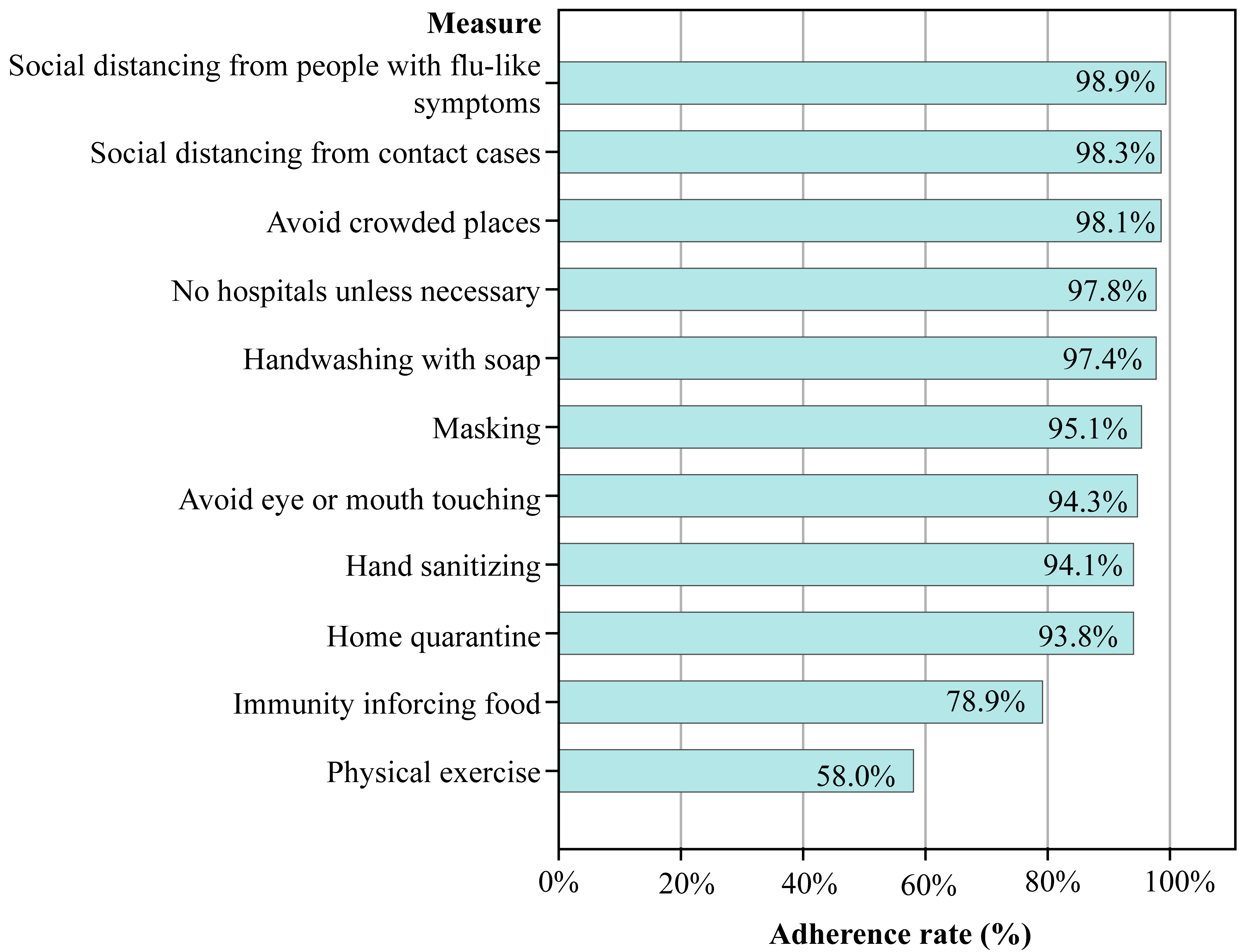

Supplement: Supplementary file 2 — Supplementary file2 Adherence levels to preventive measures against corona-virus disease (TIF 2245 KB) [file 44197_2022_83_MOESM2_ESM.tif]

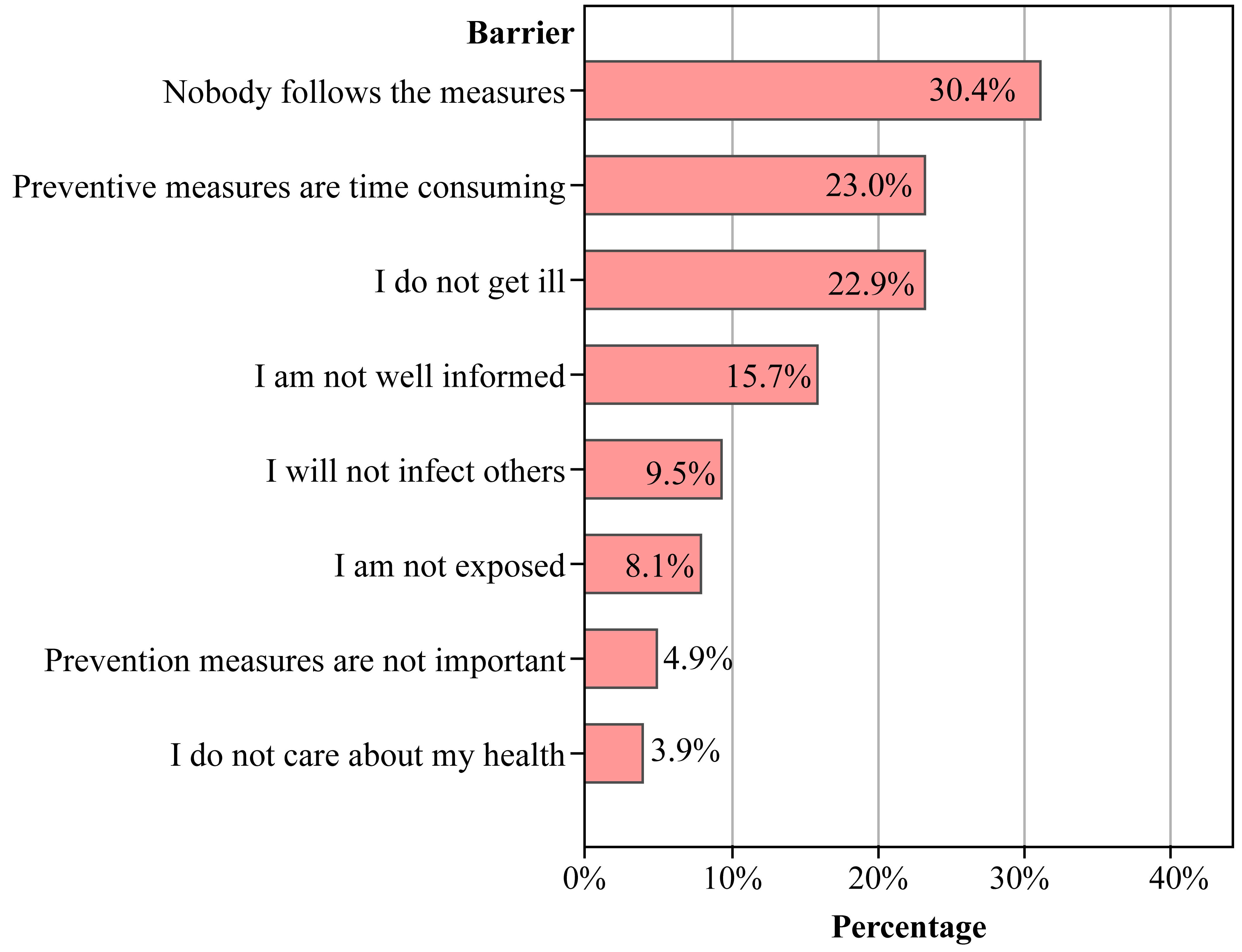

Supplement: Supplementary file 3 — Supplementary file3 Cognitive barriers to adherence to preventive measures against coronavirus disease (TIF 2089 KB) [file 44197_2022_83_MOESM3_ESM.tif]
